# An unusually prolonged course of late onset severe OHSS with successful outcome - a rare but avoidable complication

**DOI:** 10.5935/1518-0557.20210071

**Published:** 2022

**Authors:** Neena Malhotra, Anshu Yadav, Chithira Sridhar, Pankush Gupta, Monika Saini

**Affiliations:** 1 Department of Obstetrics &Gynecology. All India Institute of Medical Sciences, New Delhi, India

**Keywords:** late onset OHSS, transaminitis, hemorrhagic ascites

## Abstract

Ovarian hyperstimulation syndrome (OHSS) is an entity associated with controlled ovarian stimulation (COS) in assisted reproduction. It presents a great challenge for clinicians concerning a timely diagnosis and intervention, because it is a life-threatening condition. We came across a rare case of late onset severe OHSS, which lasted till 19 weeks of gestation (POG) with diagnostic challenge in which the patient had hemorrhagic ascites post embryo transfer, and transaminitis. The case was managed effectively with repeated paracentesis along with replacement therapy, and the transaminitis was also gradually resolved. Severe OHSS may follow an unusually prolonged course associated with transaminitis. Our case suggests that it is crucial to distinguish between the nature and etiology of transaminitis along with OHSS, and other pathologies like drug induced transaminitis should also be kept in mind.

## INTRODUCTION

Controlled ovarian stimulation (COS) is a routinely performed standard procedure in assisted reproductive technology (ART) today. One of the uncommon, but dreaded complication associated with this procedure is ovarian hyperstimulation syndrome (OHSS), which is mostly of iatrogenic origin. It can be of early onset when the exogenous hCG is given within 3 to 7 days; or late onset when the endogenous hCG is released due to early trophoblastic activity 10 to 12 days after hCG injection. Late onset OHSS tends to have more prolonged and severe course than early onset (Mathur *et al.*, 2000). It can be divided into different classes of severity depending on various clinical and laboratory parameters. We hereby, present a case report describing the course of events in a patient who developed late onset severe OHSS with hemorrhagic ascites and transaminitis after embryo transfer.

## CASE PRESENTATION

A 32 year-old female, third gravida, with a previous history of two tubal ectopic pregnancies followed subsequently by bilateral laparoscopic salpingectomies, underwent an In-Vitro fertilization cycle (IVF) with an agonist protocol and presented on 7^th^ day post embryo transfer with complaints of vomiting, abdominal pain and distension. She was admitted and evaluated.

IVF cycle details: Her Anti-mullerian hormone (AMH)-2.8 ng/dl, Antral follicle count (AFC)- 22, BMI- 22kg/m^2^, considering her tubal condition, an agonist protocol was chosen, from day 21 of the preceding cycle. Injection leuprolide acetate 0.5 mg subcutaneous (sc) was given daily for 14 days, ovarian stimulation with recombinant FSH (Gonal F- Merek Sereno) 250 IU sc daily, started on day 2 of her menses and the leuprolide dose was reduced to 0.25 mg sc, she was triggered on the 10th day of stimulation with recombinant hCG (ovidrel) 250 mcg s/c at estradiol (E2) 3137pg/ml and progesterone (P4)-1.6 ng/ml. Oocyte pickup (OPU) was done 36 hours after the trigger and 16 oocytes were retrieved. Fresh embryo transfer was done with a single blastocyst (5AA) and surplus day 3 (n=6) embryos were frozen for her.

Her baseline blood investigations upon admission revealed hemoconcentration (hemoglobin - 15.6g/dl, hematocrit - 46.8%), leucocytosis (total leucocyte count- 22,300), normal renal function tests, SGOT/SGPT/ALP- 72/98/91 U/L. Her ultrasound showed bilateral enlarged ovaries (right - 10 x 8.9 x 9cm, left - 11.1 x 9.8 x 8.4 cm) with tense ascites, largest vertical pocket 8.8 cm. So, it was labeled as late onset severe OHSS. She was admitted and started on low molecular weight heparin (LMWH) 60 mcg s.c. for thrombo-prophylaxis. Intravenous fluid replacement (crystalloids+ colloids) was initiated as standard therapy for OHSS, with strict intake output monitoring while continuing progesterone luteal support (routine luteal support with estradiol valerate 2mg twice a day, oral dydrogesterone 10 mg twice a day, intramuscular micronized progesterone 100 mg daily). Because of the severe abdominal pain and tense ascites, she was submitted to paracentesis (2 liters) on the day of admission (straw colored) and subsequently when required. On these management lines, the patient showed signs of stabilization.

On day-3 of admission, she had acute onset pallor and tense ascites. Further investigations revealed a falling hemoglobin and hematocrit. (Hb/Hct - 8.9mg/dl/26.7%) and transaminitis (Table 1). She also developed respiratory distress, subcutaneous edema and her chest X ray was suggestive of moderate left pleural effusion with basal atelectasis. As she was in the implantation phase, we did a serum beta hcg test and it was 182 mIU/ml, which rose to 365 mIU/ml after 48 hours. Repeat paracentesis was done with replacement therapy; to our surprise the ascitic fluid was bloody. She was transfused 20% albumin to supplement protein loss. Ultrasound done on the 20^th^ day post embryo transfer revealed a single live intrauterine fetus, with crown rump length 5+6 wks and good decidual reaction.

**Table 1.** Trend of blood investigations during treatment period.

Investigation	Day 0 (Adm/day)	Day 3	Day 8	Day 13	Day 18	Day 30	Day 60
**Hb (mg/dl)/HCT (%)**	15.6/46.8	7.6/23.7	7.6/23.7	9.0	9.2	10.1	10.9
**TLC (/ml)**	22,300	19,740	18,190	14,030	9,860	9,260	10,610
**Platelet (lacs)**	3.2	2.3	3.8	4.2	3.0	1.8	1.5
**Ur/Cr (mg/dl)**	20/0.5	31/0.7	12/0.5	17/0.6	16/0.5	15/0.4	10/0.4
**Na/K (mMol/L)**	143/3.9	133/5.7	141/5	134/4.8	134/4.2	135/4	136/4.5
**OT/PT/ALP (U/L)**	72/98/91	140/143/243	341/465/538	576/654/626	378/528/488	106/120/310	32/39/244
**TP/A/G (g/dl)**	4.8/2.8/2.0	4.2/2.6/1.6	5.2/3/2.0	4.6/2.1/2.5	5.5/3.3/2.2	6/3.1/2.9	6.8/3.9/3.9
**T.bil (mg/dl)**	0.5	0.6	0.6	0.5	0.3	0.4	0.2

### Investigations

Table 1 shows her blood workup during the treatment period.

### Differential diagnosis

The extraordinary persistence of OHSS was a differential diagnostic problem, like her exceptional liver transaminitis with hemorrhagic ascites on the day 3 of admission, and an unusual hemodilution as compared to hemoconcentration usually seen with OHSS. Therefore, several diagnostic imaging studies and specific laboratory tests were ordered to rule out the possibility of other serious disorders and portal hypertension as a cause. Transaminitis showed an increasing trend with advancing gestation, along with leucocytosis raised the suspicion of sepsis, portal hypertension and iatrogenic liver injury as the cause.

### Treatment

With repeated paracentesis and volume replacement she improved. She required albumin transfusion (20%) twice in view of her hypoalbuminemia. LMWH was continued till 19 weeks POG. Transaminitis also decreased. She improved and was discharged under stable condition after seven days of hospital stay.

### Outcome and follow-up

She was kept on a strict and regular follow up. The visiting schedule was twice weekly for one month and then once-a-week till 19 weeks of gestation (POG). Paracentesis was done as required clinically during the follow up visits. In total she was tapped of 32 liters of ascites in a total of 16 sittings. Her transaminitis resolved gradually after discontinuation of estradiol valerate, after 2 weeks since presentation. Her hemoglobin also rose over a period of 4 weeks without any blood transfusion and the color of her ascitic tap also reverted back to the original straw color. Afterwards, follow-up ultrasound examinations showed resolution of her ascites, normalization of ovary size, her subsequent fetal development was satisfactory, and laboratory parameters also normalized. Her antenatal follow up with growth scans was normal. She underwent elective cesarean section on maternal request at 39 weeks POG and delivered a baby boy, of 3200 grams. Intraoperatively there was around 200 ml ascitic fluid and her post-operative course was uneventful.

## DISCUSSION

OHSS is a potentially life-threatening complication associated with COS in IVF cycles (Whelan & Vlahos, 2000; Practice Committee of the American Society for Reproductive Medicine, 2004). Some amount of transaminitis is associated with moderate to severe OHSS. The reason for the elevated transaminases remains speculative and a field of interest. One of the possible causes may be the destruction of hepatocytes as a result of the supraphysiological estrogen concentrations. However, in a study by Forman *et al.* (1990) no significant correlation was found between the maximum estradiol concentration and the severity of transaminitis. But because of the concurrent occurrence of elevated aminotransferases with ascites (Younis *et al*., 1988; Balasch *et al*., 1990; Ryley *et al*., 1990), the involvement of the renin-angiotensin-aldosterone system, together with increased vessel permeability and hepatic edema in severe OHSS (Navot *et al.,* 1987) may be the cause of hepatic cell destruction (Wakim & Fox, 1996). Balasch *et al*. (1990) described on a patient with severe OHSS together with drug-induced cholestasis.

The case reported herein is remarkable for the unusually prolonged course of late onset severe OHSS (till 19 weeks POG), as well as for exceptional liver transaminitis with hemorrhagic ascites and unusual hemodilution, as compared to the hemoconcentration usually seen with OHSS. Unrelenting manifestations of ovarian hyperstimulation persisted despite standard therapy and the progression of gestational age. Therefore, several diagnostic imaging studies and specific laboratory tests were made to exclude the possibility of other serious disorders and portal hypertension as a cause. Procalcitonin and ultrasound Doppler were found normal, and gastroenterology consultation was sought because they found it to be drug-induced and asked to stop estradiol valerate and oral dydrogesterone along with micronized progesterone. That was a big challenge to us because withdrawing all the luteal support simultaneously at such early gestation, i.e. 5 weeks, was a difficult task, so we planned to do it in a sequential way by curtailing the most hepatotoxic first, i.e. estradiol and oral dydrogestone, followed by replacement of intramuscular micronized progesterone with vaginal progesterone gel to decrease systemic absorption. With the repeated paracentesis and the gradual withdrawal of luteal support, the patient started improving, frequency of taps reduced from daily initially to twice weekly and then weekly. Also her liver transaminases showed a decreasing trend and came back to normal, her hemoglobin level also improved to normal without any transfusion or iron supplementation, and above all the color of the ascitic fluid reverted to straw colored.

## CONCLUSION

In our case, the patient behaved as a high responder. An antagonist protocol with a agonist trigger and freeze all strategy would have been the ideal option for her. So, better protocol planning is the crux of ART but with timely diagnosis and intervention keeping the mind open for other diagnostic possibilities, we can have a successful outcome even in challenging situations like this one.
